# Seven year review of retention in HIV care and treatment in federal medical centre Ido-Ekiti

**DOI:** 10.11604/pamj.2015.22.139.4981

**Published:** 2015-10-14

**Authors:** Oluwoleadeyemi Babatunde, Olujide John Ojo, Oladele Ademola Atoyebi, David Sylvanus Ekpo, Adebusuyi Opeyemi Ogundana, Temitope Oluseun Olaniyan, John Adeyemi Owoade

**Affiliations:** 1 Department of Community Medicine, Federal Medical Center, PMB 201, Ido-Ekiti, Nigeria; 2 Care and Support Unit, Department of Community Medicine, Federal Medical Center, PMB 201, Ido-Ekiti, Nigeria; 3 Department of Community Medicine, Obafemi Awolowo University Teaching Hospital Complex, Ile-Ife, Nigeria

**Keywords:** AIDS, antiretroviral, CD4, HIV, retention

## Abstract

**Introduction:**

Poor retention of patients in care is a major driver of poor performance and increased morbidity and mortality in HIV/AIDS programme despite the expansion and advancement Anti-retroviral Therapy (ART). The objective of this study is to assess retention rates and possible determining factors in People Living with HIV (PLHIV) on ART.

**Methods:**

This is a descriptive, cross-sectional study conducted in Federal Medical Center, Ido-Ekiti, Nigeria. Medical records of clients who were enrolled in ART Care and support unit (HIV Clinic) of the health facility from 2005 to 2012 were reviewed and analyzed using SPSS version 16. A total of 621 client records were reviewed for basic demographic information, CD4 count, WHO stage, number of follow-up visit, client ART status and client retention status (defined as client attending at least one clinic visit in 2012.

**Results:**

A total of 347(63%) patients were retained in care and 208(37%) were not retained over the seven year review period. Retention was statistically significant with age (P-value 0.031), ART status (P-value 0.000) baseline CD4 (P-value 0.004), year of diagnosis and ART initiation (P-value= 0.027). Poor retention was associated decreasing age, pre-ART client, HIV stage 1&IV client and baseline CD4 above 400cell/mm^3^.

**Conclusion:**

Retention in care of PLHIV is a minimum necessary condition for maintaining or restoring health in the long run. The strategies to sustain and improve retention rate should be adopted to maximize ART benefits. A follow-up study on other factors affecting retention from diagnosis to long term retention ART programme is recommended.

## Introduction

For Human Immuno-deficiency Virus (HIV)-infected patients, retention in care is a minimum necessary condition for maintaining or restoring health. Of the 33 million persons living with Human Immuno-deficiency Virus/Acquired Immune Deficiency Virus (HIV/AIDS), 23 million reside in sub-Saharan Africa, nearly 5 million in Asia, and 1.6 million in Latin America and the Caribbean. This implies that the burden of HIV infection is highest in sub-Saharan Africa. The remarkable expansion of access to ART for HIV/AIDS in resource-constrained countries has given about seven million HIV-positive adults in sub-Saharan Africa the opportunity to achieve what for many may be nearly normal life expectancies [1]. Despite significant success in scaling up ART programmes worldwide, many people living with HIV (PLHIV) start ART late in the progression of HIV infection, resulting in high rates of early mortality on ART [2]. The majority of PLHIV remain undiagnosed and many do not access HIV care and treatment despite a positive test. They are lost at every step along the continuum of care, particularly in the period between HIV diagnosis and initiation of ART. It is now recognized that poor retention of patients in care, especially in the pre-ART period is a major driver of poor performance and increased morbidity and mortality in HIV/AIDS programme [2]. Retention in HIV care can be defined as continuous engagement from the time of diagnosis in a package of prevention, treatment, support and care services. It is defined from the moment of initial engagement in care, when a person with HIV is linked successfully to services, to assessment for eligibility, initiation on ART and retention in lifelong ART care [3]. Retention in HIV care is also the ability to adhere to critical aspects of care, attend regular follow-up appointments, scheduled lab tests, and other monitoring activities, according to health system standards and as prescribed by a health care provider [4]. Retention is critical to reduce HIV-related morbidity and mortality, reduce the incidence of new infections in children and adults, and reduce development of ART resistance. Different schools of thought have defined retention in care in terms of number of clinical visit at given time frame. Three studies published in 2002 measured patient retention rates by using roughly the same definition of 1 medical visit every 6 months over a 2-year period [5–7]. A wide range was found in the retention rates (18%-61%). Another study done in Nigeria, measured patient retention as having one or more clinic visits in the review year [8].

Retention in care has been highlighted as an important element of clinical success for the patient and the program. Whereas attending clinical appointments is associated with favorable patient outcomes among individuals with HIV on ART [9, 10]. Poor retention in care has been associated with higher mortality for both ART and pre-ART patients in both high-income and resource-limited settings [11–13]. A study from Kenya found that patients not retained in care are generally sicker than those who are retained in care and may therefore experience poorer long-term outcomes [14]. In addition to retrieving medication, clinical follow-up visits are crucial for monitoring drug toxicity, clinical HIV progression, and to diagnose and treat new opportunistic infections (OIs) and other concurrent diseases that may occur [4]. Identifying patients who are not retained in care can be challenging as poor retention can include a range of behaviors such as missing a single scheduled clinical visit to lost-to-follow-up (LTFU), a term use to describe patients who fail to present to clinic for a certain period of time and are not known to have died [13]. Although overall treatment adherence among sub-Sahara African patients has been high, recent evidence suggests that a large number of PLHIV in the region who have started in treatment programs are not retained in care. A review of 33 patient cohorts taking ART in 13 African countries suggested only 60 percent of patients remain enrolled in programs after two years, with LTFU accounting for 56 percent of all attrition [13]. Furthermore, a study conducted in Uganda found that over 25 percent of patients eligible for ART did not complete screening or begin treatment [11]. The potentially high attrition rates suggest the need for a better understanding of how PLHIV integrate ART and care seeking behavior in the context of their daily lives to support adherence to treatment and program retention.

The proportion of adult patients retained between any two points from testing positive for HIV to initiating ART in sub-Saharan African HIV/AIDS care programs were categorized in 3 stages; Stage 1 (from HIV testing to receipt of CD4 count results or clinical staging), Stage 2 (from clinical staging to ART eligibility) an Stage 3 (from ART eligibility to ART initiation). The range reported for the proportions of patients retained in Stage 1 was 35%-88% and a median range of 59%. For Stage 2 the range was 31%-95% with a median range of 46%. While for Stage 3 the range was 14%-84% and the median range 68% [15]. One key reason for the poor retention of pre-ART clients in care is persistence of low starting CD4 counts for ART eligibility which results in failure of linking HIV positive clients from HIV testing (stage 1) to HIV care and retention in care. As most patients are asymptomatic during the pre-ART period, they may not perceive themselves as requiring medical care hence lack understanding in the importance of enrolling in care. Other reasons for poor retention include issues of disclosure like fear being recognized as a client of an HIV clinic. Issues of stigmatization and discrimination that could result in job lost. Patient mobility due to factors like severe illness, distance to clinic, lack of transportation and Poverty [16, 17]. Those presented with low CD4 count are likely to have died before reaching stage of ART. Some health facility factors that affect retention include poor attitude of health workers, lack of good organization. Long waiting time at facilities may also be a factors contributing to low retention rates [11, 18, 19]. Although some estimates suggest that retention in care is poor in Africa; around 50% to 70% at 2 years after ART initiation -these estimates rely on studies that consider patients as retained in care only when they are retained in a particular clinic; this is an unrealistic expectation in a decentralized system and in regions where people frequently relocate to maintain a livelihood. Studies that attempt to account for patients as they move across the network of care suggest retention rates are considerably higher, up to 85%-90% at 2 years [20, 21]. This study was conducted to assess retention rates and possible determining factors in PLHIV on ART in the care and support unit of Federal Medical Centre, Ido-Ekiti, Ekiti State, south-west Nigeria.

## Methods

This is a descriptive cross-sectional study, conducted in Federal Medical Center (FMC), Ido-Ekiti which is one of the two tertiary health institutions in Ekiti state, South-west, Nigeria. The primary data were obtained from medical records of clients who were enrolled in ART Care and support unit (HIV Clinic) of the health facility from 2005 to 2012. The care and support unit was commenced in 2005 as integration of normal hospital care and metamorphosed into a full fleshed HIV programme in 2010. Basic demographic information, CD4 count, WHO stage, number of follow-up visit, client ART status and client retention status (defined as client attending at least one clinic visit in year 2012) were reviewed and analyzed. A total of 621 client records were reviewed and entered into a performer, then into a computer software SPSS version 16.0. All persons tested positive to HIV but not enrolled were excluded from the study. Patients that died before year of review were not considered for retention. Client outcomes in care were assessed as “in care”, “loss to follow-up” “discontinued care” and “died”. SPSS version 16.0 was used to analyses the data. A trend analysis of clinical and outcome data as well as their association with treatment outcomes were done. Chi-square test was used determining factors associated with retention. A p-value of less than 0.05 was considered as statistically significant. Ethical approval for the study was obtained from the ethical and review committee of Federal Medical Centre, Ido-Ekiti. The limitation of this study was that for patient who did not give valid address or phone contact, determining their retention status in terms of whether transferred out or died was difficult.

## Results

**Figure 1:**
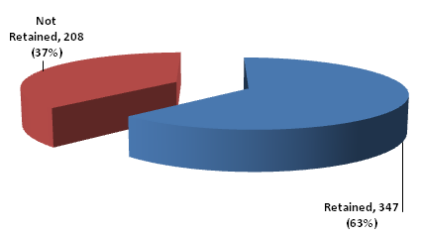
Distribution of patients’ retention status

**Figure 2:**
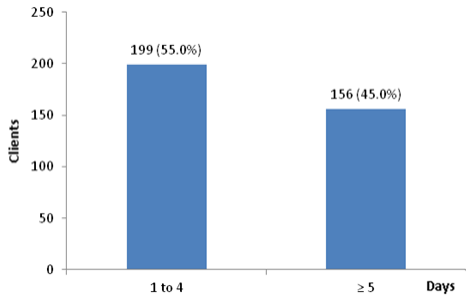
Number of clinic days among the retained clients

The study population included 621 patients; 426(68.6%) were female and 195 (31%) were males (Table 1) The mean age at time of enrollment was 36. 33 ± 13.12years. With age 26 to 49 accounting for over two-third (71.2%) of patients as seen in Table 1. A larger percentage patient 65.5% were diagnosed from 2009 to 2011 and 64.2% initiating ART within the same year (Table 2). During the period of study, a total of 531(87%) were on ART while 80 (12.9%) had not initiated ART. At initiation of ART, 54.3% of the patients had CD4 ≤200 cells/mm^3^. Three hundred and fifty two patients (56.7%) at diagnosis were in WHO stage 3 and 4. One hundred and nine (17.6%) in stage 2, while 160(25.8%) in stage 1. In the year of review 2012, a total of 274(44.1%) patient had zero clinic attendance, 191(30.8%) attended clinic between 1-4 times. While, 156(25.1%) had greater than 5 clinic visits. Figure 1 shows the retention status of patient; a total of 347 (63%) were retained while 208(37%) where not retained over a seven year period (Figure 2)(Table 3). The rate of retention per year was highest in the first year 2005 with five enrollees and with all of them retained that year followed by 2010 and 2006 with rates of 73 and 72.3 percent respectively. In 2008 and 2011 retention rate were below average with 48.4 and 49.7 percent respectively. Table 4 shows the factors affecting retention. There is statistically significant association between age and retention status (P-value of 0.031). Retention increased with age; it was lowest in age ≤15 years (42.9%) and highest in age ≤50 years (65. 3%). Sex was not statistically significant with retention (P- value of 0.307). ART status showed a strong association with retention (P-value of 0.000). More than two- third (67.3%) of PLHIV on ART were retained, while only 20.3% of those not on ART were retained. The CD4 count at diagnosis with P- value of 0.004 was statistically significant with higher retention on patient with CD4 bellow 400 and lower in those with CD4 above 400. The retention rate was 72.8% from 2005 to 2007, 57.5% from 2008 to 2009 and 61.5% from 2010 to 2011. Therefore the year of diagnosis was statistically significant with rate of retention at a P-value of 0.027. Year of initiation on ART was also statistically significant as the year of diagnosis with similar P-value of 0.027(Table 5). There was no statistically significant association between level of retention with age, sex, ART status, CD4 count, WHO staging at diagnosis, year of diagnosis and year of ART initiation as shown in Table 5.

**Table 1:** Year of diagnosis, art initiation and socio- demographic characteristics of HIV clients

Variable		N = 621	
		Frequency	Percentage
**Age Group (in years)**			
	< 15	43	6.9
	15 – 19	2	0.3
	20 – 25	43	6.9
	26 – 49	442	71.2
	≥ 50	91	14.7
	Mean = 36.33±13.123		
**Sex**			
	Female	426	68.6
	Male	195	31.4
**Year of Diagnosis**			
	2005 – 2006	34	5.5
	2007 - 2008	180	29.0
	2009 – 2011	407	65.5
**Year of ART Initiation**			
	2005 – 2006	31	5.7
	2007 – 2008	163	30.1
	2009 – 2011	347	64.2

**Table 2:** WHO staging and clinic attendance of HIV clients

Variable		N = 621	
		Frequency	Percentage
**ART Status**			
	Yes	541	87.1
	No	80	12.9
**CD4 Count**			
	≤ 100	196	31.6
	101 – 200	141	22.7
	201 – 300	99	15.9
	301 – 400	74	11.9
	>400	111	17.9
**WHO Stage at Diagnosis**			
	1	160	25.8
	2	109	17.6
	3	185	29.8
	4	167	26.9
**Number of Clinic Days Attended**			
	0	274	44.1
	1 – 4	191	30.8
	≥5	156	25.1

**Table 3:** Percentage of patients retained per year

Year	Number Enrolled	Number Retained in 2012	Percentage Retention
2005	5	5	100.0
2006	29	21	72.4
2007	89	57	64.1
2008	91	44	48.4
2009	102	60	58.8
2010	124	75	73.5
2011	181	90	49.7
Total	621	352	56.7

**Table 4:** Factors affecting retention rate

		Retention		X^2^	p – value
		Yes	No		
**Age**	<15	18 (42.9)	24 (57.1)	**10.672**	**0.031**
	15 – 19	1 (50.0)	1 (50.0)		
	20 - 25	20 (51.3)	19 (48.7)		
	26 – 49	259 (65.2)	138 (34.8)		
	≥50	49 (65.3)	26 (34.7)		
**Sex**				**1.044**	**0.307**
	Female	243 (63.9)	137 (36.1)		
	Male	104 (59.4)	71 (40.6)		
**ART Status**				**50.135**	**0.000**
	Yes	335 (67.5)	161 (32.5)		
	No	12 (20.3)	47 (79.7)		
**CD4 Count**				**15.500**	**0.004**
	≤100	101 (66.9)	50 (33.10		
	101 – 200	75 (59.5)	51 (40.5)		
	201 – 300	69 (72.6)	26 (38.1)		
	301 – 400	48 (66.7)	24 (33.3)		
	>400	54 (48.6)	57 (51.4)		
**WHO Stage at Diagnosis**				**5.666**	**0.129**
	1	94 (59.1)	65 (40.9)		
	2	76 (72.4)	29 (27.6)		
	3	99 (61.9)	61 (38.1)		
	4	78 (59.5)	53 (40.5)		
**Year of Diagnosis**				**7.234**	**0.027**
	2005 – 2007	83 (72.8)	31 (27.2)		
	2008 – 2009	104 (57.5)	77 (42.5)		
	2010 – 2011	160 (61.5)	100 (38.5)		
**Year of Initiation**				**7.234**	**0.027**
	2005 – 2007	83 (72.8)	31 (27.2)		
	2008 – 2009	104 (57.5)	77 (42.5)		
	2010 – 2011	160 (61.5)	100 (38.5)		

**Table 5:** Level of retention among clients

		Level of Retention		X^2^	p – value
		1 – 4	≥5		
**Age**	<15	14 (77.8)	4 (22.2)	**4.852**	**0.303**
	15 – 19	1 (100.0)	0 (0.0)		
	20 – 25	11 (55.0)	9 (45.0)		
	26 – 49	139 (53.7)	120 (46.3)		
	**≥**50				
**Sex**				**4.852**	**0.303**
	Female	136 (56.0)	107 (44.0)		
	Male	55 (52.9)	49 (47.1)		
**ART Status**				**0.054**	**0.816**
	Yes	184 (54.9)	151 (45.1)		
	No				
**CD4 Count**				**3.223**	**0.521**
	≤100	55 (54.5)	46 (45.5)		
	101 – 200	46 (61.3)	29 (38.7)		
	201 – 300	33 (47.8)	36 (552.2)		
	301 – 400	25 (52.1)	23 (47.9)		
	>400	32 (59.3)	32 (40.7)		
**WHO Stage of Diagnosis**				**2.462**	**0.482**
	1				
	57 (60.6)	37 (39.4)			
	2	42 (55.3)	34 (44.7)		
	3	54 (54.5)	45 (45.50		
	4	38 (48.7)	40 (51.3)		
**Year of Diagnosis**				**2.340**	**0.310**
	2005 – 2007	14 (53.8)	12 (46.2)		
	2008 – 2009	62 (61.4)	39 (38.6)		
	2010 – 2011	115 (52.3)	105 (47.7)		
**Year of Initiation**				**2.340**	**0.310**
	2005 – 2007	14 (53.8)	12 (46.2)		
	2008 – 2009	62 (61.4)	39 (38.6)		
	2010 – 2011	115 (52.3)	105 (47.7)		

## Discussion

Retention in long-term HIV care both before and after the initiation of ART is important not only to reduce individual HIV-related mortality and morbidity but also as a means to deliver “positive prevention” interventions aimed at reducing ongoing transmission, In this seven years study of retention among HIV patient in a tertiary health institution, south-west Nigeria, approximately only 3 in 5 (63%) patients were retained while 37% were not retained between March 2005 and December 2012. Among those retained, 335(67.5%) patient were on ART while only 12(20.3%) were Pre-ART patient, after eliminating those who died or whose records could not be found, from the study. This was very similar to findings from other HIV treatment programme; Ugoji et al. [8] which showed 62.4% retention; out of which 75.8% of patients were on ART, and 23.4% were not on ART. Studies from 2007-2009 from across Africa, estimated a median retention in care for 12 months after treatment initiation to be 79.4%, with a range of 55-93% [2]. In a meta-analysis of the pre-ART attrition in Sub-Saharan Africa [22], the attrition in Stage 1 was 22.4% and the attrition in Stage 3 was 37.1%. In a single site cohort study in South Africa [23], the cumulative retention in care of ART eligible patients from HIV diagnosis to 6-12 months after ART initiation was 36.9%. Although these studies differ in some of the stage definitions and follow-up periods, the rates of attrition are similar to our study, suggesting comparable rates of attrition in Sub-Saharan Africa and India. The poor retention rate in pre-ART patient is due to CD4 count greater than 350 cell/mm3 and absence of illness that would have warranted their visiting the hospital. Lessells et al., in their study in South Africa showed that the increase ART initiation to 350cell/mm3 in 2010 guide line, improved retention rates [23]. In our analysis, increased risk of LTFU or poor retention was associated with age, CD4 level at ART initiation, ART status, year of diagnosis and year of initiation of ART. Younger age was associated with poor retention. In this study, retention increased with age; it was lowest in age ≤15 years (42.9%) and highest in age ≤50 years (65. 3%). Similarly two studies in 2011 and 2012, by Indian National AIDS Control Organization [22, 24], showed that younger adults fail to access health services efficiently. Studies by Lessells et al., Forster et al., Bassett et al., Bateganya et al., Wanyenze et al. and Helleringer et al. were also consistent with findings [23, 25–29]. The WHO report on retention in 2011 stated that, diagnosing and retaining HIV-exposed and infected children and adolescents in care present unique challenges [1]. Their vulnerability is heightened by dependence upon a caregiver. The CD4 count was statistically significant in our study with P-value of 0.004. There was high retention rate in patient with baseline CD4 bellow 400cell/mm^3^ while those with baseline CD4 greater than 400cell/mm^3^ had poor retention rate. This was consistent with studies by Lessells et al. and Charurat et al. [23, 30]. Although retention was lowest in baseline CD4 < 100cells/mm^3^ and > 350cell/mm^3^ in study by Charurat et al, it was a high (66.9%) in patient with CD4 less than 100cell/mm^3^ in our study. However in a study by Alvarez-Uria et al, there was no significant association between baselines CD4 cell counts above 350cell/mm^3^ with poor retention [31].

Retention rates decreased with years of enrollment because more people live the programme from reasons of death, change in location, feeling of wellness after vial load have been suppressed, improved conditions of health or other economic and religious factors, especially in Africa. According to data presented by Stevenson at a Conference on Retroviruses and Opportunistic Infections (CROI) in Seattle, aggregate six-month non-retention rates were 21 percent in 2005 and 2010, and there was no significant difference in the 12 month non-retention rates in 2005 or 2010 - which were 27 and 29 percent respectively [32]. However Cote d'Ivoire, Nigeria and Rwanda had increased non-retention rates during this period. There was no significant association between retention with gender and WHO staging at diagnosis. Although in this study female patient were about two-third (68.6%) of study subject and had slightly marginal retention rate than men, there was no statistically significantly associated between male or female (P- value 0.309 (>0.05)). In many other study reviewed there was higher probability for women to be retain in HIV programmes than men [13, 30, 33, 34]. The finding that higher proportion of women initiated on ART remained in follow-up, may reflect gender-differences in health-seeking behaviors which has been shown to affect retention in care in other resource-limited countries [35, 36]. WHO staging was not a significant risk factor to the retention rate in our study, with a P-value of 0.129(>0.05), retention was highest in patient with WHO stage 2 (72%) than stages 1, 3, 4 with marginal difference of 1.8%. However the study by Ugoji et al. showed a strong association between retention and WHO staging as retention decreased with higher baseline WHO stage [8]. There was no statistical significant difference between the levels of retention in those retained with factors like age, sex, ART status, CD4 count WHO staging at diagnoses and year of diagnosis or ART initiation.

## Conclusion

Retention in care of PLHIV is a minimum necessary condition for maintaining or restoring health in the long run. With the scaling of ART programme and the expansion of PLHIV on medication especially in low and middle income countries, drastic measures and strategy should be deployed to address the issue of long term retention in care. This is to maximize the gains and sustain progress already made, halt and reverse HIV/AIDS pandemic in line with achieving MDG goal 6. The predictors of long time retention in HIV programmes are age, ART status, and baseline CD4 count, year of diagnosis and year of ART initiation. This also corresponds to findings in many other studies reviewed, with varying methods and design. Though our study was limited by the challenges of missing records and difficulty in tracing whether some of the patients were dead, transferred out, discontinued care or actually loss to follow-up. We recommend that quality data management in HIV programmes in health facility should be strengthen to improve results from researches that will improve the strategies and procedures to boost long term retention. A follow-up analytical or experimental study will be conducted to highlight other factors associated long term retention from HIV testing to linkage in care and long term continuum in care.
